# Identification of key DNA methylation changes on fasting plasma glucose: a genome-wide DNA methylation analysis in Chinese monozygotic twins

**DOI:** 10.1186/s13098-023-01136-4

**Published:** 2023-07-17

**Authors:** Weijing Wang, Wenqin Yao, Qihua Tan, Shuxia Li, Haiping Duan, Xiaocao Tian, Chunsheng Xu, Dongfeng Zhang

**Affiliations:** 1 Department of Epidemiology and Health Statistics, School of Public Health, Qingdao University, No. 308 Ningxia Road, Qingdao, 266071 Shandong Province China; 2 Shandong Province Center for Disease Control and Prevention, Shandong, China; 3 Epidemiology and Biostatistics, Department of Public Health, University of Southern Denmark, Odense, Denmark; 4 Qingdao Municipal Center for Disease Control and Prevention/Qingdao Institute of Preventive Medicine, Qingdao, Shandong China

**Keywords:** Causality, DNA methylation, Fasting plasma glucose, Monozygotic twins

## Abstract

**Background:**

Elevated fasting plasma glucose (FPG) levels can increase morbidity and mortality even when it is below the diagnostic threshold of type 2 diabetes mellitus (T2DM). We conducted a genome-wide DNA methylation analysis to detect DNA methylation (DNAm) variants potentially related to FPG in Chinese monozygotic twins.

**Methods:**

Genome-wide DNA methylation profiling in whole blood of twins was performed using Reduced Representation Bisulfite Sequencing (RRBS), yielding 551,447 raw CpGs. Association between DNAm of single CpG and FPG was tested using a generalized estimation equation. Differentially methylated regions (DMRs) were identified using *comb-P* approach. ICE FALCON method was utilized to perform the causal inference. Candidate CpGs were quantified and validated using Sequenom MassARRAY platform in a community population. Weighted gene co-expression network analysis (WGCNA) was conducted using gene expression data from twins.

**Results:**

The mean age of 52 twin pairs was 52 years (SD: 7). The relationship between DNAm of 142 CpGs and FPG reached the genome-wide significance level. Thirty-two DMRs within 24 genes were identified, including *TLCD1*, *MRPS31P5*, *CASZ1*, and *CXADRP3*. The causal relationship of top CpGs mapped to *TLCD1*, *MZF1*, *PTPRN2*, *SLC6A18*, *ASTN2*, *IQCA1*, *GRIN1*, and *PDE2A* genes with FPG were further identified using ICE FALCON method*.* Pathways potentially related to FPG were also identified, such as phospholipid-hydroperoxide glutathione peroxidase activity and mitogen-activated protein kinase p38 binding. Three CpGs mapped to *SLC6A18* gene were validated in a community population, with a hypermethylated direction in diabetic patients. The expression levels of 18 genes (including *SLC6A18* and *TLCD1*) were positively correlated with FPG levels.

**Conclusions:**

We detect many DNAm variants that may be associated with FPG in whole blood, particularly the loci within *SLC6A18* gene. Our findings provide important reference for the epigenetic regulation of elevated FPG levels and diabetes.

**Supplementary Information:**

The online version contains supplementary material available at 10.1186/s13098-023-01136-4.

## Introduction

Type 2 diabetes mellitus (T2DM) is a chronic metabolic disease with a high prevalence characterized by chronic hyperglycemia, which can cause serious complications, such as heart attack, blindness, and nerve and blood vessel damage. As an important indicator for T2DM diagnosis, elevated fasting plasma glucose (FPG) levels can increase morbidity and mortality even when it is below the diagnostic threshold [1].4

The FPG levels and T2DM may be influenced by a combination of genetic factors and environmental exposure with being mediated by epigenetic modification [2]. At present, the magnitude of genetic sources of variance in FPG and T2DM has been extensively explored. The heritability of FPG has been reported to range from 24.90% to 67.66% [3–5]. Additionally, some genome-wide association studies (GWASs) have reported the genetic variants responsible for susceptibility to elevated FPG levels and T2DM, such as the genetic loci in/near *SPATS2L*, *SLC26A11*, and *JAZF1* [2, 3]. However, the genetic variants identified in previous GWASs just could explain less than 20% of the estimated heritability for T2DM [6] and thus could only partially contribute to the pathogenesis of this disease.

In recent years, increasing evidence has supported the significant role of epigenetic mechanisms with altered gene expression in the increased susceptibility to diseases. DNA methylation (DNAm) is an important aspect of epigenetic research, and several existing epigenome-wide association studies (EWASs) have investigated the relationship of DNAm with T2DM and glycemic traits [7–18]. Although some cytosine phosphate guanines (CpGs) and genes have been reported, replicated CpGs and genes are limited. For example, Walaszczyk et al. previously identified 52 T2DM-related CpGs in peripheral blood, however, only 5 CpGs located at *LOXL2*, *TXNIP*, *SLC1A5*, *SREBF1* and *ABCG1* were replicated after strict multiple corrections [11]. Thus, more EWASs on T2DM or glycemic traits are needed for further replication and validation. In addition, given that most of the reported associations are from cross-sectional and case–control designs, the causal nature of the relationship, that is, if DNAm exerts a causal effect on FPG or vice versa, is unknown. Therefore, it is necessary to further explore the causal relationship between DNAm and FPG.

The limited detection and replication of CpGs and genes among EWASs may be due to the use of unrelated individuals as controls in traditional cross-sectional [9, 13, 14] or case–control studies [7, 8, 10–12, 17]. Although common factors such as age, sex and race were fully considered, the confounding effect from different genetic backgrounds was not well controlled [19]. Nowadays, monozygotic twins with the same genetic background have been proved to be ideal samples for EWASs [20]. Especially for the modest and highly heritable traits or diseases, the use of monozygotic twins in EWAS can improve statistical power by perfectly controlling for the effect of different genetic background [21]. Nevertheless, currently only a few studies have investigated the effect of epigenetics on T2DM or glycemic traits in monozygotic twins [15, 18]. Furthermore, the use of monozygotic twins also makes causal inference possible in association studies of epigenetics based on cross-sectional design [22]. To our knowledge, no studies have yet performed causal inference analysis between them.

The Chinese population may have different DNAm variants compared to other ethnic groups owing to the different genetic makeup and environmental exposure. However, to data, the EWASs on T2DM or glycemic traits performed in Chinese population were limited, particularly in twins [23]. Herein, we conducted this EWAS to explore the potential CpGs, genes and biological pathways potentially related to FPG in Chinese monozygotic twins, and further estimated the causation between DNAm variants and FPG. Candidate CpGs were further validated in a community population. Finally, we integrated the differentially methylated results with gene expression data in twins.

## Methods

The primary materials and methods used in this study were in accordance with those of our previously published studies [24–29].

### Participants

Monozygotic twin samples were collected through the Qingdao twin registry, and details of study recruitment have been previously described [30]. We excluded participants who were pregnant or breastfeeding, had used hypoglycemic drugs or insulin, and did not complete a questionnaire or physical examination. The participants had unqualified blood samples, such as blood collection vessel ruptured, or the concentration or total amount of extracted DNA could not meet the experimental requirements, were further dropped. Moreover, incomplete twin pairs that either of the twins lacked blood sample or relevant information were also excluded. Considering the advantage of trait or disease-discordant monozygotic twin design, the particularity of monozygotic twin samples, and our experience in previous research [24–29], the twins with intra-pair FPG difference ≥ 0.1 mmol/L was chosen. A total of 52 complete monozygotic twin pairs were included in the methylation analysis, and a subsample of 12 pairs were randomly selected for gene expression analysis. The median of intra-pair absolute difference of FPG (|Δ(FPG)|) in all twins was 0.48 mmol/L (95% range: 0.13–1.80).

FPG levels were determined using a semiautomatic analyzer. Sex, ABO blood type, and 16 multiple short tandem repeat DNA markers were used to identify zygosity. Informed consent was obtained from all the participants. Ethical approval was obtained, and the study was conducted in accordance with the Declaration of Helsinki.

### Reduced representation bisulfite sequencing (RRBS) analysis and data preprocessing

The DNA extracted from whole blood was sent to a corporation (Biomarker Biological Technology, Beijing, China) for RRBS analysis. Briefly, genomic DNA was treated with MspI to generate short fragments containing CpG dinucleotides. Then, end-repair, dA-tailing, and purification processes were performed to obtain CpG-rich DNA fragments. The obtained DNA fragments were bisulfite-converted, amplified by polymerase chain reaction (PCR), and sequenced using Illumina HiSeq X Ten. The raw methylation data covered 551,447 CpGs across the genome of each individual.

The pipeline recommended by Bismark was adopted to preprocess raw data [31]. Sequencing data were aligned to the Genome Reference Consortium Human Build 37 using Bowtie2 [32]. The processed coverage outputs were then inputted to R package *BiSeq* to generate a smooth methylation level [33]. To reduce bias, we limited the coverage to 90% quantile and further removed CpGs with an average methylation *β*-value < 0.05 or more than ten missing observations. After quality control, a total of 252,564 CpGs remained for subsequent analyses. The methylation *β*-value was converted to *M*-value with M = log_2_(*β*/(1- *β*)) for further analysis [34].

### Cell-type composition estimation

Considering that DNAm data was measured in whole blood, distinct methylation profiles of different cell types may give rise to false discoveries [35]. The *ReFACTor* method was introduced to attenuate the effects of distinct cell components on DNAm [36]. Specifically, *ReFACTor* selected methylation sites that provided important information on cell composition for principal component analysis (PCA). The top five components of PCA were used to structure the underlying substitutes of cell type composition to adjust the heterogeneity of cell types.

### Construction, sequence, and quality control of RNA library

The detailed experimental process has been described in our previous study [25]. Briefly, mRNA was extracted from whole blood using TRIzol reagent, and its concentration, purity, and integrity were rigorously measured. After purification, fragment size selection, and PCR enrichment, the qualified mRNA was used to construct the RNA-seq library. The RNA-seq library was then sequenced to obtain sequencing data using the Illumina HiSeq 2500 and was validated by real-time quantitative PCR (RT-qPCR). The TopHat2 was used to map the sequencing data to the human genome [37]. The FPKM value was used to detect gene expression levels using Cufflinks software [38].

### Statistical analysis

#### Epigenome-wide association analysis

The association between DNAm *M*-value at a single CpG and FPG was tested by applying generalized estimation equation (GEE) approach through *geeglm* function in R-package *geepack*, adjusting for age, sex, diastolic blood pressure (DBP) and top five cell-type composition. Furthermore, in order to address the paired structure of twin data, we included a vector which identified the clusters of twins within a pair into the GEE model. To take multiple testing into account, we calculated the false discovery rate (FDR) [39] and defined genome-wide significance as FDR < 0.05. The identified genomic CpGs (*P* < 0.05) were annotated to the nearest genes using R-package *biomaRt* [40].

#### Detecting differentially methylated regions (DMRs)

The DMRs associated with FPG were evaluated using *Comb-P* approach that could calculate auto-correlation, combine adjacent *P*-values, performe false discovery adjustment, find regions of enrichment, and assign significance to those regions successively [41]. The Stouffer-Liptak-Kechris (*slk*) corrected *P*-value < 0.05 was used to detect significantly enriched DMRs.

#### Causal inference analysis

For the identified top 30 CpGs, the causal relationship with FPG was estimated by Inference about Causation through Examination of Familial Confounding (ICE FALCON) method in twins [22]. In this method, ‘familial’ meant both genetic and shared environmental factors in twins, which was essential for making explicit causal inference. The GEE model was applied for parameter estimation with correction for twin pairing. Estimations of *β*_self_, *β*_co-twin_, as well as *β′*_self_, and *β′*_co-twin_ were calculated, where *β*_self_ was the estimation of the overall correlation including the causal proportion and family confounding proportion, *β*_co-twin_ estimated only the family confounding proportion of the correlation, and *β′*_self_ and *β′*_co-twin_ was the estimation of the full model. If |*β*_co-twin_*–β′*_co-twin_| was similar to |*β*_self_*–β′*_self_|, then the association was due to family confounding; and if |*β*_co-twin_*–β′*_co-twin_| was much larger than |*β*_self_*–β′*_self_| (ratio > 1.5), then it indicated a causal effect [42].

#### Ontology enrichment analysis

In order to further explore the biological function of CpGs identified in EWAS, we submitted 20,925 CpGs (*P*-value < 0.05) to the Genomic Regions Enrichment of Annotations Tool (GREAT) online to analyze ontology enrichments [43]. Annotation was based on human GRCh37, and the default “basal plus extension” association rule was used. Statistical significance was defined as FDR < 0.05.

### Sensitivity analysis

In order to evaluate the robustness of study findings, we performed a sensitivity analysis by further adjusting for smoking status (now or ever smoking versus never smoking) and drinking status (now or ever drinking versus never drinking) in the original GEE model in epigenome-wide association analysis. Subsequently, the DMRs were also explored, and causal inference analysis was also performed. We also performed another sensitivity analysis by removing the outliers for DNAm of each top CpG and then testing the association between DNAm of each CpG and FPG again.

#### Power of epigenome-wide association analysis

We have published a computer simulation study on the power of EWAS using twin design [21]. According to this study, for one trait/disease with a heritability of 0.6, the sample size required for the statistical power to exceed 80% in the twin design ranged from 22 to 63 pairs when the correlation between environmental factors and DNA methylation ranged from 0.8 to 0.1, which is an immense improvement over the ordinary case–control design. The heritability of FPG was about 0.68 in Chinese twins [3]. Hence, our study, based on 52 twin pairs, would get a statistical power of about 80%.

#### Quantitative methylation analysis of SLC6A18

Considering the results of top DNAm signals identified in EWAS, the biological function of genes, the causal relationship with FPG, the correlation of gene expression level with FPG, and the primers designed results, we selected the *SLC6A18* gene to validate in the community population. In the case–control study, we randomly recruited 72 diabetic cases and 170 healthy controls from the community, with no restrictions or criteria on the selection of the controls. The patients were defined as those with a fasting FPG level ≥ 7.0 mmol/L, taking hypoglycemic drugs, or using insulin. Participants with a history of hypertension, obesity, cancer, stroke, cardiovascular disease, or hepatitis were excluded. The participants were interviewed, and blood samples were collected and stored at -80^◦^C for DNA methylation analysis.

We designed primers for *SLC6A18* gene to cover the region with the most CpGs (*P*-value < 0.05) in EWAS. The mass spectra of the cleavage products were collected using MALDI-TOF mass spectrometry based on the MassARRAY System (Bio Miao Biological Technology, Beijing, China), and the methylation ratio of the spectra was generated using MassARRAY EpiTYPER software (Agena Bioscience, San Diego, California, USA). The DNAm of CpGs and characteristics between the two independent groups was compared using Wilcoxon rank sum test or *t* test. A binary logistic regression model was applied to evaluate the association between CpGs and T2DM while adjusting for total cholesterol (CHOL), triglyceride (TG), and low-density lipoprotein cholesterol (LDLC). Statistical significance was set at *P*-value < 0.05.

#### Weighted gene co-expression network analysis (WGCNA)

In order to investigate whether the genes where the top CpGs and DMRs were mapped in methylation analysis were also differentially expressed, we further performed a weighted gene co-expression network analysis (WGCNA) using the gene expression data of twins. The R-package *WGCNA* is a comprehensive function that can perform weighted correlation network analysis [44, 45]. Briefly, first, a weighted adjacency matrix was established. Then, we constructed a topological overlap matrix (TOM) and used it as an input for conducting hierarchical clustering analysis. A dynamic tree-cutting algorithm was used to detect the gene modules. The module eigengenes (MEs) of the modules were correlated with FPG levels.

Furthermore, in order to find the important biological function on FPG and diabetes, we also performed functional enrichment analysis for the genes clustered in modules related to FPG and tried to find the common enrichment terms between methylation analysis and gene expression analysis. The BIOCARTA, KEGG, and REACTOME pathway enrichment analysis and GO enrichment analysis were performed using DAVID tool for genes clustered in important modules [46]. A modified Fisher’s exact *P*-value < 0.05 was treated as the cut-off standard for significant enrichments.

## Results

### Epigenome-wide association analysis

In this study, 52 twin pairs (including 27 male pairs) with a mean age of 52 years (SD: 7) were included. The median FPG level was 5.44 mmol/L (95% range: 3.76–7.4). Most clinical indicators showed a statistically significant correlation in twin pairs (Additional file 1: Table S1), suggesting the co-twin design beneficial. However, the correlation of DBP (*r* = 0.207, *P* = 0.141) showed no statistical significance, hence we treated DBP as a covariate in the subsequent GEE model.

The Manhattan plot is shown in Additional file 2: Fig. S1. The association between DNAm of 142 CpGs and FPG reached the genome-wide significance level (FDR < 0.05). The top 30 CpGs are shown in Table 1. The strongest association (*β* = 2.49, FDR = 1.80 × 10^–5^) was determined for the CpG (chr12:105,478,501 bp) in *ALDH1L2*. These top CpGs were located in/near 17 genes, such as *TLCD1*, *SYNPO*, *MZF1*, *PTPRN2*, *SLC6A18*, *ASTN2*, and *IQCA1*.

**Table 1:** The results of DNA methylation of top 30 CpGs with fasting plasma glucose.

CpG No.	Chromosome	Position (bp)	*β*	*P*-value	FDR	HGNC symbol
1	chr12	105,478,501	2.488	7.11E−11	1.80E−05	*ALDH1L2*
2	chr17	27,052,829	2.717	8.37E−09	6.20E−04	*TLCD1*
3	chr19	658,314	0.319	1.17E−08	6.20E−04	*RNF126*
4	chr5	150,027,611	0.248	1.21E−08	6.20E−04	*SYNPO*
5	chr19	59,073,819	0.308	1.23E−08	6.20E−04	*MZF1*^a^
6	chr7	157,670,224	0.228	1.55E−08	6.52E−04	*PTPRN2*^a^
7	chr17	27,052,816	2.723	2.26E−08	7.92E−04	*TLCD1*
8	chr8	26,148,178	− 1.772	2.72E−08	7.92E−04	*PPP2R2A*
9	chr19	59,073,806	0.261	3.03E−08	7.92E−04	*MZF1*^a^
10	chr5	150,027,616	0.240	3.14E−08	7.92E−04	*SYNPO*
11	chr17	27,052,798	2.778	3.53E−08	8.11E−04	*TLCD1*
12	chr17	27,052,771	2.780	9.88E−08	2.08E−03	*TLCD1*
13	chr5	1,233,035	0.221	1.40E−07	2.70E−03	*SLC6A18*^a^
14	chr5	1,233,066	0.181	1.61E−07	2.70E−03	*SLC6A18*^*a*^
15	chr5	1,233,041	0.216	1.65E−07	2.70E−03	*SLC6A18*^a^
16	chr5	1,233,045	0.210	1.71E−07	2.70E−03	*SLC6A18*^a^
17	chr19	59,073,831	0.338	1.93E−07	2.87E−03	*MZF1*^a^
18	chr9	119,332,867	0.324	2.44E−07	3.43E−03	*ASTN2*^a^
19	chr9	119,332,835	0.306	3.30E−07	4.39E−03	*ASTN2*^a^
20	chr7	157,670,239	0.200	4.07E−07	5.02E−03	*PTPRN2*^a^
21	chr9	133,911,755	0.123	4.18E−07	5.02E−03	*LAMC3*
22	chr2	237,298,168	-2.138	4.52E−07	5.19E−03	*IQCA1*
23	chr2	237,298,161	-2.126	4.76E−07	5.23E−03	*IQCA1*
24	chr3	129,059,046	-2.528	5.34E−07	5.62E−03	*MARK2P19*
25	chr5	29,364,034	0.232	7.16E−07	7.05E−03	*LINC02064*
26	chr10	126,490,028	0.177	7.26E−07	7.05E−03	*FAM175B*
27	chr9	140,033,560	− 0.277	8.28E−07	7.44E−03	*GRIN1*^a^
28	chr18	14,458,789	− 0.513	8.52E−07	7.44E−03	*LONRF2P1*
29	chr5	1,233,018	0.225	8.73E−07	7.44E−03	*SLC6A18*^a^
30	chr11	72,352,936	0.232	8.84E−07	7.44E−03	*PDE2A*^a^

NA, not available; *β*, regression coefficient

^a^Genes already suggested by previous studies

### Differentially methylated regions (DMRs) analysis

**Fig. 1:**
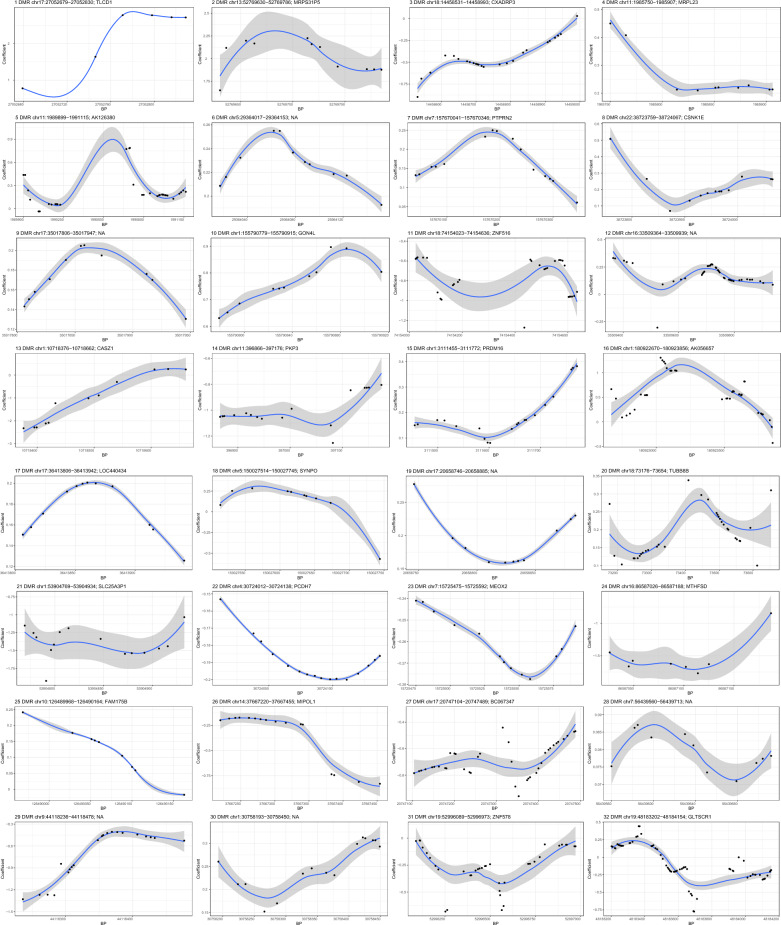
The methylation patterns for the identified differentially methylated regions (DMRs). The horizontal axis shows the chromosome positions with the black point indicating each CpG, and the vertical axis shows the coefficient for the association between each CpG and fasting plasma glucose (FPG). Black line indicates the methylation pattern for each DMR. BP, base pair; chr, chromosome. NA, not available

As shown Fig. 1 and Table 2, a total of 32 DMRs were identified for FPG. The methylation level of 18 DMRs (1, 2, 4–10, 12, 15–17, 19, 20, 25, 28, and 30) at/near *TLCD1*, *MRPS31P5*, *MRPL23*, *AK126380*, *PTPRN2*, *CSNK1E*, *GON4L*, *PRDM16*, *AK056657*, *LOC440434*, *TUBB8B*, and *FAM175B* was positively associated with FPG level, and 12 DMRs (3, 11, 13, 14, 21–24, 26, 27, 29, and 31) at/near *CXADRP3*, *ZNF516*, *CASZ1*, *PKP3*, *SLC25A3P1*, *PCDH7*, *MEOX2*, *MTHFSD*, *MIPOL1*, *C067347*, and *ZNF578* was negatively associated with FPG level, respectively. However, the association between the methylation level of two DMRs (18 and 32) and FPG level was difficult to determine. Four DMRs covered several of the top CpGs as depicted in Table 1, with DMR25 (*FAM175B*) covering one CpG, DMR7 (*PTPRN2*) and DMR18 (*SYNPO*) covering two CpGs, and DMR1 (*TLCD1*) covering four CpGs, respectively.

**Table 2:** The results of annotation to significant differentially methylated regions

DMR No.	Chromosome	Start	End	Length	*slk* corrected *P*-value	Gene symbol
1	chr17	27,052,679	27,052,830	6	3.76E−09	*TLCD1*^a^
2	chr13	52,769,630	52,769,786	11	2.36E−08	*MRPS31P5*^a^
3	chr18	14,458,531	14,458,993	25	2.78E−07	*CXADRP3*
4	chr11	1,985,750	1,985,907	10	1.36E−07	*MRPL23*^a^
5	chr11	1,989,899	1,991,115	33	2.42E−06	*AK126380*
6	chr5	29,364,017	29,364,153	11	3.46E−07	NA
7	chr7	157,670,041	157,670,346	15	1.07E−06	*PTPRN2*
8	chr22	38,723,759	38,724,067	13	3.11E−06	*CSNK1E*
9	chr17	35,017,806	35,017,947	11	1.82E−06	NA
10	chr1	155,790,779	155,790,915	11	2.43E−06	*GON4L*
11	chr18	74,154,023	74,154,636	30	1.25E−05	*ZNF516*
12	chr16	33,509,364	33,509,939	41	1.27E−05	NA
13	chr1	10,718,376	10,718,662	12	8.32E−06	*CASZ1*^a^
14	chr11	396,866	397,176	17	9.04E−06	*PKP3*
15	chr1	3,111,455	3,111,772	20	1.02E−05	*PRDM16*
16	chr1	180,922,670	180,923,856	41	4.87E−05	*AK056657*
17	chr17	36,413,806	36,413,942	12	5.79E−06	*LOC440434*
18	chr5	150,027,514	150,027,745	10	1.43E−05	*SYNPO*
19	chr17	20,658,746	20,658,885	11	8.99E−06	NA
20	chr18	73,176	73,654	32	4.12E−05	*TUBB8B*
21	chr1	53,904,769	53,904,934	15	1.55E−05	*SLC25A3P1*
22	chr4	30,724,012	30,724,138	16	1.21E−05	*PCDH7*
23	chr7	15,725,475	15,725,592	13	1.21E−05	*MEOX2*
24	chr16	86,587,026	86,587,188	8	1.70E−05	*MTHFSD*
25	chr10	126,489,968	126,490,164	9	2.19E−05	*FAM175B*
26	chr14	37,667,220	37,667,455	16	2.91E−05	*MIPOL1*
27	chr17	20,747,104	20,747,489	38	5.03E−05	*BC067347*
28	chr7	56,439,560	56,439,713	11	2.06E−05	NA
29	chr9	44,118,236	44,118,478	21	3.85E−05	NA
30	chr1	30,758,193	30,758,450	15	4.50E−05	NA
31	chr19	52,996,089	52,996,973	37	1.70E−04	*ZNF578*
32	chr19	48,183,202	48,184,154	68	1.88E−04	*GLTSCR1*

DMR, differentially methylated region; Length, the number of CpGs located in each DMR; NA, not available

^a^Genes already suggested by previous studies

### Causal inference analysis

The causal inference results of the top CpGs are depicted in Table 3. Briefly, the DNAm of seven CpGs located at/near three genes (*SLC6A18*, *IQCA1*, and *PDE2A*) was bidirectionally associated with FPG, that was, when FPG changed DNAm changed, and vice versa. The DNAm changes at eight CpGs (*MZF1*, *PTPRN2*, *GRIN1*, and *SLC6A18*) caused FPG changes, and FPG changes caused DNAm changes at five other CpGs (*TLCD1* and *ASTN2*). The causal relationship between the remaining CpGs and FPG was not statistically significant.

**Table 3:** The results of causal inference analysis of top CpGs with fasting plasma glucose

CpG No.	Chromosome	Position	HGNC symbol	Methylation to FPG					FPG to methylation				
				*β*_self__ _change_	*P*_self__ _change_	*β*_co-twin__ _change_	*P*_co-twin__ _change_	Absolute value of ratio	*β*_self__ _change_	*P*_self__ _change_	*β*_co-twin__ _change_	*P*_co-twin__ _change_	Absolute value of ratio
1	chr12	105,478,501	*ALDH1L2*	0.016	4.63E−10	0.018	2.89E−10	1.065	− 0.500	3.13E−01	− 0.645	2.10E−01	1.290
2^**b**^	chr17	27,052,829	*TLCD1*	0.017	2.03E−06	0.024	3.94E−05	1.421	**− 0.329**	**5.35E−01**	**− 1.059**	**1.90E−02**	**3.218**
3	chr19	658,314	*RNF126*	0.046	1.40E−03	0.068	1.86E−06	1.472	− 0.023	6.84E−01	− 0.170	1.56E−01	7.489
4	chr5	150,027,611	*SYNPO*	0.082	1.23E−03	0.105	2.20E−03	1.283	− 0.024	6.25E−01	− 0.049	2.60E−01	2.090
5^a^	chr19	59,073,819	*MZF1*	**0.029**	**3.96E−01**	**0.151**	**9.44E−03**	**5.230**	0.018	7.19E−01	− 0.114	7.82E−02	− 6.429
6^a^	chr7	157,670,224	*PTPRN2*	**0.077**	**3.46E−06**	**0.150**	**2.03E−09**	**1.931**	0.001	9.81E−01	− 0.072	1.12E−01	− 71.878
7^**b**^	chr17	27,052,816	*TLCD1*	0.016	7.29E−05	0.023	5.30E−04	1.449	**− 0.366**	**4.68E−01**	**− 1.077**	**1.80E−02**	**2.945**
8	chr8	26,148,178	*PPP2R2A*	− 0.017	7.86E−05	−0.011	2.21E−03	0.618	0.630	1.23E−01	0.133	7.52E−01	0.212
9^a^	chr19	59,073,806	*MZF1*	**0.022**	**5.99E−01**	**0.112**	**4.71E−02**	**5.141**	0.010	8.42E−01	− 0.080	1.76E−01	− 7.954
10	chr5	150,027,616	*SYNPO*	0.075	3.24E−03	0.095	4.41E−03	1.257	− 0.023	6.43E−01	− 0.044	3.23E−01	1.936
11^**b**^	chr17	27,052,798	*TLCD1*	0.014	6.90E−04	0.019	2.95E−03	1.345	**− 0.448**	**3.91E−01**	**− 0.963**	**4.57E−02**	**2.149**
12	chr17	27,052,771	*TLCD1*	0.013	5.05E−03	0.015	1.54E−02	1.159	− 0.568	3.03E−01	− 0.797	1.41E−01	1.404
13^a, **b**^	chr5	1,233,035	*SLC6A18*	**0.140**	**7.45E−09**	**0.253**	**1.90E−09**	**1.808**	**0.005**	**8.90E−01**	**− 0.091**	**1.38E−02**	**− 18.774**
14^a, **b**^	chr5	1,233,066	*SLC6A18*	**0.091**	**4.87E−06**	**0.177**	**2.09E−10**	**1.950**	**0.003**	**9.41E−01**	**− 0.070**	**3.82E−02**	**− 25.124**
15^a, **b**^	chr5	1,233,041	*SLC6A18*	**0.134**	**8.86E−08**	**0.250**	**9.29E−09**	**1.862**	**0.008**	**8.17E−01**	**− 0.093**	**1.18E−02**	**− 11.256**
16^a, **b**^	chr5	1,233,045	*SLC6A18*	**0.134**	**1.61E−07**	**0.249**	**1.14E−08**	**1.867**	**0.009**	**8.00E−01**	**− 0.092**	**1.09E−02**	**− 10.206**
17^a^	chr19	59,073,831	*MZF1*	**0.025**	**4.45E−01**	**0.150**	**4.51E−03**	**6.074**	0.024	6.57E−01	− 0.130	8.19E−02	− 5.416
18^**b**^	chr9	119,332,867	*ASTN2*	0.154	2.04E−06	0.204	9.23E−08	1.331	**− 0.044**	**4.75E−01**	**− 0.159**	**3.81E−03**	**3.636**
19^**b**^	chr9	119,332,835	*ASTN2*	0.091	1.65E−03	0.129	1.24E−05	1.425	**− 0.047**	**4.56E−01**	**− 0.121**	**3.26E−02**	**2.598**
20^a^	chr7	157,670,239	*PTPRN2*	**0.048**	**3.30E−03**	**0.162**	**5.31E−09**	**3.402**	0.019	6.17E−01	− 0.070	1.01E−01	− 3.636
21	chr9	133,911,755	*LAMC3*	− 0.030	5.49E−01	0.088	1.59E−01	− 2.905	0.018	6.28E−01	− 0.029	3.65E−01	− 1.556
22^a, **b**^	chr2	237,298,168	*IQCA1*	**− 0.016**	**1.05E−06**	**−0.026**	**2.19E−07**	**1.626**	**0.0001**	**1.00E + 00**	**1.064**	**1.11E−02**	**7362.729**
23^a, **b**^	chr2	237,298,161	*IQCA1*	**− 0.016**	**1.11E−06**	**−0.026**	**2.24E−07**	**1.628**	**− 0.003**	**9.95E−01**	**1.064**	**1.13E−02**	**− 407.723**
24^a^	chr3	129,059,046	NA	**− 0.001**	**9.16E−01**	**−0.022**	**6.60E−03**	**41.613**	− 0.326	4.63E−01	0.935	5.72E−02	− 2.869
25	chr5	29,364,034	*LINC02064*	0.044	3.14E−02	0.062	2.76E−03	1.419	− 0.030	6.24E−01	− 0.146	2.55E−01	4.921
26	chr10	126,490,028	*FAM175B*	− 0.036	6.57E−01	0.209	9.03E−02	− 5.886	0.010	7.41E−01	− 0.028	4.45E−01	− 2.713
27^a^	chr9	140,033,560	*GRIN1*	**0.029**	**2.49E−01**	**−0.047**	**4.72E−02**	**− 1.618**	− 0.055	2.76E−01	0.064	1.29E−01	− 1.165
28	chr18	14,458,789	*LONRF2P1*	− 0.001	9.69E−01	−0.023	3.46E−01	18.309	− 0.027	8.78E−01	0.121	3.42E−01	− 4.420
29^a^	chr5	1,233,018	*SLC6A18*	**0.099**	**3.60E−04**	**0.193**	**3.06E−06**	**1.951**	0.0003	9.93E−01	− 0.078	6.70E−02	− 223.473
30^a, **b**^	chr11	72,352,936	*PDE2A*	**0.143**	**1.95E−06**	**0.253**	**5.51E−10**	**1.770**	**− 0.006**	**8.45E−01**	**− 0.088**	**4.89E−03**	**14.239**

Results in bold indicate the causation statistically significant

NA, not available; FPG, fasting plasma glucose

^a^The change of DNA methylation causes the FPG change

^b^The FPG change causes the change of DNA methylation

### Ontology enrichments analysis

Important pathways potentially related to FPG and diabetes were found, including phospholipid-hydroperoxide glutathione peroxidase activity, mitogen-activated protein kinase p38 binding, positive regulation of insulin receptor signaling pathway, cell fate commitment, notch signaling pathway, and biosynthesis of neurotransmitters (Additional file 3: Table S2).

#### Sensitivity analysis

Additionally, we performed a sensitivity analysis by further adjusting for smoking status and drinking status in the original GEE model in epigenome-wide association analysis. The smoking and drinking status of intra-pair twins were almost consistent in twin samples. The numbers of twins with both smoking, both non-smoking, and inconsistent smoking status were 19, 28, and 5, respectively. The numbers of twins with both drinking, both non-drinking, and inconsistent drinking status were 17, 32, and 3, respectively. As shown in Additional file 4: Table S3, Additional file 5: Table S4, and Additional file 6: Table S5, the results of epigenome-wide association analysis, DMRs analysis, as well as causal inference analysis were almost consistent with those before sensitivity analysis, indicating our findings robust. Moreover, when we removed the outliers for DNAm of each top CpG in another sensitivity analysis, the association between DNAm of each CpG and FPG remained nearly constant.

### Quantitative methylation analysis of SLC6A18

A total of 72 diabetic cases and 170 healthy controls from the community were included to validate the CpGs mapped to *SLC6A18* gene identified in EWAS. As shown in Additional file 7: Table S6, people with diabetes had higher levels of CHOL, higher levels of TG, and lower levels of LDLC than people without diabetes. Of the CpGs identified (*P*-value < 0.05) mapped to *SLC6A18* in EWAS, three were quantified using the Sequenom MassARRAY platform in a community population. As shown in Additional file 8: Table S7, these three CpGs were hypermethylated in T2DM patients, showing a same direction as in EWAS. Particularly, one CpG (chr5:1,233,066) was also regarded as the top DNAm signal shown in Table 1.

### WGCNA

Twelve twin pairs were included in gene expression analysis, with a mean age of 54 years (SD: 6) and a median FPG level of 5.60 mmol/L (95% range: 4.31–7.90). As shown in Additional file 9: Fig. S2, the Darkolivegreen module (including 721 genes) was positively correlated with FPG levels (*r* = 0.61, *P*-value = 0.001).

Among the genes where the top CpGs and DMRs were mapped in methylation analysis, 18 genes were also clustered in the Darkolivegreen module of WGCNA (Additional file 10: Table S8), including *ALDH1L2*, *TLCD1*, *RNF126*, *SYNPO*, *MZF1*, *PTPRN2*, *PPP2R2A*, *SLC6A18*, *ASTN2*, *LAMC3*, *IQCA1*, *FAM175B*, *GRIN1*, *PDE2A*, *MRPL23*, *CASZ1*, *GON4L*, and *CSNK1E*. Moreover, some common enrichment terms were also identified, such as extracellular matrix structural constituent, dopamine binding, dopamine neurotransmitter receptor activity, regulation of biosynthetic process, neuron fate specification, and C21-steroid hormone biosynthetic process (Additional file 11: Table S9).

## Discussion

Based on monozygotic twin samples, we identified some CpGs, DMRs, and pathways potentially associated with FPG. Three CpGs mapped to *SLC6A18* gene were also validated in a community population. Common genes and enrichment terms between the DNA methylation and gene expression analyses were identified. In addition, causal relationship between DNAm of several CpGs and FPG was identified. What’ more, the results of sensitivity analysis indicated our findings robust.

Some genes where the top CpGs and DMRs were mapped may influence FPG levels or diabetes, such as *MZFI*, *PTPRN2*, *ASTN2*, *GRIN1*, *SLC6A18*, and *PDE2A*. The *MZFI* gene binds and transactivates L-selectin promoter, which has been proven to be related to disease entities, including T2DM [47]. While comparing the mouse data with T2DM patients data, altered DNAm of 105 genes was correlated with incident T2DM, among which *PTPRN2* showed a stronger predictive potential [8]. The association between genetic variants of *ASTN2* gene and T2DM has been determined [48, 49]. It was reported that SNP rs6293 in *GRIN1* gene could affect eating behavior in T2DM [50]. The protein encoded by *SLC6A18* gene is a member of the *SLC6* family, which acts as a transporter for small molecules, including alpha-D-glucose. The *SLC6A18* gene is involved in the proximal tubule transport pathway, and insulin might participate in renal glucose handling by acting on the proximal tubules [51]. In addition, the *SLC6A18* gene is also involved in the NRF2 pathway, and its role in mastering antioxidants in diabetic dysfunction has been clearly elucidated [52]. Early and specific upregulation of cardiac *PDE2A* gene expression was noted in diabetic cardiomyopathy [53]. Other genes currently have unknown roles in FPG levels or diabetes, and they may be potential candidates for further exploration and validation.

Additionally, when we integrated the DNA methylation data with gene expression data, we found that the gene expression levels of several genes where the top CpGs and DMRs were mapped in methylation analysis was positively correlated with FPG levels. It is worth noting that the roles of *PTPRN2*, *ASTN2*, *GRIN1*, *SLC6A18*, and *PDE2A* in influencing FPG levels or diabetes had previously been suggested as mentioned above. We speculated that the DNA methylation variants in these genes might influence FPG levels by regulating the corresponding gene expression levels. Moreover, we also found some common enrichment terms between methylation analysis and gene expression analysis, such as extracellular matrix structural constituent [54], dopamine binding and dopamine neurotransmitter receptor activity [55, 56], and C21-steroid hormone biosynthetic process [57], which might play important roles in the elevated FPG levels and diabetes and might serve as important targets to be further researched.

As additional replication, we compared previously reported significant results in a series of EWASs with ours. Due to the different methods to assess methylation profiles, we mainly compared the results among studies based on the genes where the significant CpGs were mapped. As shown in Additional file 12: Table S10, three differentially methylated genes including *C7orf50*, *PTPRN2*, and *CASZ1* could be replicated. The association between methylated levels of *C7orf50* gene and T2DM or glycemic traits has previously been reported in Koreans [7], Saharan African [9], Britons [10], Americans [16], and Chinese [18], as well as in a meta-analysis of five prospective European cohorts [58]. Ouni et al. found that *PTPRN2* gene showed a stronger predictive potential for T2DM [8]. Abnormal DNAm levels at the promoter of *CASZ1* gene in the placental may lead to metabolic diseases, including T2DM [59]. All of these indicated that our results are credible.

Three strengths in our study can be noted. First, our study was the implementation of a trait-discordant monozygotic twin model, which is proven to be a powerful tool for EWAS [20, 21]. supporting the relevance of our results. Second, causal relationships between the DNAm of some top CpGs and FPG was identified. Third, considering the differences in genetic background and environmental exposure, the underlying pathogenic process of diabetes in Chinese population may be partly referred to in our study.

However, several limitations of this study cannot be ignored. First, compared to the traditional case–control design, the sample size of our study was relatively limited because of the challenges of recruiting and identifying high-quality monozygotic twins. However, according to a study on the power of EWAS using a twin design [21], our study based on 52 twin pairs would obtain a statistical power of about 80%. Moreover, the CpGs mapped to *SLC6A18* gene were successfully validated in a community population. Second, this study was based on peripheral venous blood samples from twins. The development of diabetes is disrupted by multiple biological mechanisms in different organs of the body, including the pancreas, skeletal muscle, and adipose tissue [60]. The EWASs conducted in these tissues may provide a comprehensive understanding of the etiology of diabetes. However, such tissue samples cannot be obtained on a large scale. Given the relative availability and potential value of peripheral venous blood samples in population methylation studies, most EWASs are now performed using whole blood [61]. Third, given that the eligible twins were limited, we did not perform the analysis by sex.

## Conclusions

Multiple methylated CpGs, DMRs, crucial genes (particularly *SLC6A18*), and biological pathways potentially related to FPG were identified. Our findings provide reference for the epigenetic regulation of elevated FPG levels and diabetes.

## Supplementary Information

**Additional file 1: Table S1**. Basic characteristics of the participants.

**Additional file 2: Figure S1**. Circular Manhattan plot for epigenome-wide association study on fasting plasma glucose. The numbers of chromosome and the -log_10_ of *P*-values for statistical significance are shown. Dots represent the observed CpGs.

**Additional file 3: Table S2**. The top GREAT ontology enrichments potentially related to fasting plasma glucose using binomial test.

**Additional file 4: Table S3**. The results of DNA methylation of top 30 CpGs with fasting plasma glucose in epigenome-wide association analysis in sensitivity analysis that further adjusting for smoking status and drinking status in GEE model.

**Additional file 5: Table S4**. The results of annotation to significant differentially methylated regions for fasting plasma glucose in sensitivity analysis.

**Additional file 6: Table S5**. The results of causal inference analysis of top CpGs with fasting plasma glucose in sensitivity analysis.

**Additional file 7: Table S6**. Basic characteristics of participants from the community in the quantitative methylation analysis of SLC6A18 gene

**Additional file 8: Table S7**. Validation analysis results for the CpGs mapped to SLC6A18 gene.

**Additional file 9: Figure S2**. Relationships between consensus module eigengenes and external trait. Each row in the table corresponds to a consensus module, and each column to a trait. Numbers in the table report the correlations of the corresponding module eigengenes and trait with the *P*-values printed below the correlations in parentheses. The table is color coded by correlation according to the shade of color legend. FPG, fasting plasma glucose.

**Additional file 10: Table S8**. The common genes between DNA methylation analysis and gene expression analysis.

**Additional file 11: Table S9**. The common biological enrichment terms between DNA methylation analysis and gene expression analysis.

**Additional file 12: Table S10**. Comparison between our results and other previously reported fasting plasma glucose-associated differentially methylated genes where significant CpGs were mapped.

## Data Availability

The datasets used and/or analyzed during the current study are available from the corresponding author on reasonable request.

## Abbreviations

CpG: Cytosine phosphate guanine
DBP: Diastolic blood pressure
DMR: Differentially methylated region
EWAS: Epigenome-wide association study
FDR: False discovery rate
FPG: Fasting plasma glucose
GREAT: Genomic regions enrichment of annotations tool
GWAS: Genome-wide association study
GEE: Generalized estimation equation
PCA: Principal component analysis
RRBS: Reduced representation bisulfite sequencing
SNPs: Single nucleotide polymorphisms
T2DM: Type 2diabetes mellitus
WGCNA: Weighted gene co-expression network analysis
