# Supplementary material for: Identification of key DNA methylation changes on fasting plasma glucose: a genome-wide DNA methylation analysis in Chinese monozygotic twins
Source: Diabetol Metab Syndr. 2023 Jul 17;15:159. doi: 10.1186/s13098-023-01136-4 (PMC10351111; doi:10.1186/s13098-023-01136-4)
Supplement: Supplementary file 1 — Additional file 1: Table S1. Basic characteristics of the participants. [file 13098_2023_1136_MOESM1_ESM.docx]

**Additional file 1: Table S1**. Basic characteristics of the participants.

| **Characteristics** | **Values** | **Intrapair correlation** | |
| --- | --- | --- | --- |
|  |  | ***r*** | ***P*-value** |
| Number of twin pairs | 52 |  |  |
| Sex, pairs (%) |  |  |  |
| Male | 27 (51.92) | - | - |
| Female | 25 (48.08) | - | - |
| Age, years | 52.12 (7.44) | - | - |
| FPG, mmol/L | 5.44 (3.76, 7.4) | 0.599 | < 0.001 |
| BMI, kg/m^2^ | 24.81 (3.27) | 0.477 | < 0.001 |
| Systolic, mmHg | 130 (101.25, 186.13) | 0.392 | 0.004 |
| Diastolic, mmHg | 82 (62, 109) | 0.207 | 0.141 |
| SUA, μmol/L | 294 (147.38, 550.63) | 0.604 | < 0.001 |
| CHOL, mmol/L | 5.08 (2.59, 6.69) | 0.490 | < 0.001 |
| TG, mmol/L | 1.14 (0.18, 3.38) | 0.490 | < 0.001 |
| HDLC, mmol/L | 1.36 (0.72, 2.71) | 0.776 | < 0.001 |
| LDLC, mmol/L | 2.83 (0.85) | 0.419 | 0.002 |

**Note:** Continuous variables were presented as mean (standard deviation (SD)) or median (P_2.5_, P_97.5_); Categorical variables were presented as numbers with percentages. *r*, correlation coefficient; BMI, body mass index; CHOL, total cholesterol; FPG, fasting plasma glucose; HDLC, high-density lipoprotein cholesterol; LDLC, low-density lipoprotein cholesterol; SUA, serum uric acid; TG, triglyceride.
