# Supplementary material for: Identification of key DNA methylation changes on fasting plasma glucose: a genome-wide DNA methylation analysis in Chinese monozygotic twins
Source: Diabetol Metab Syndr. 2023 Jul 17;15:159. doi: 10.1186/s13098-023-01136-4 (PMC10351111; doi:10.1186/s13098-023-01136-4)
Supplement: Supplementary file 2 — Additional file 2: Figure S1. Circular Manhattan plot for epigenome-wide association study on fasting plasma glucose. The numbers of chromosome and the -log10 of P-values for statistical significance are shown. Dots represent the observed CpGs. [file 13098_2023_1136_MOESM2_ESM.docx]

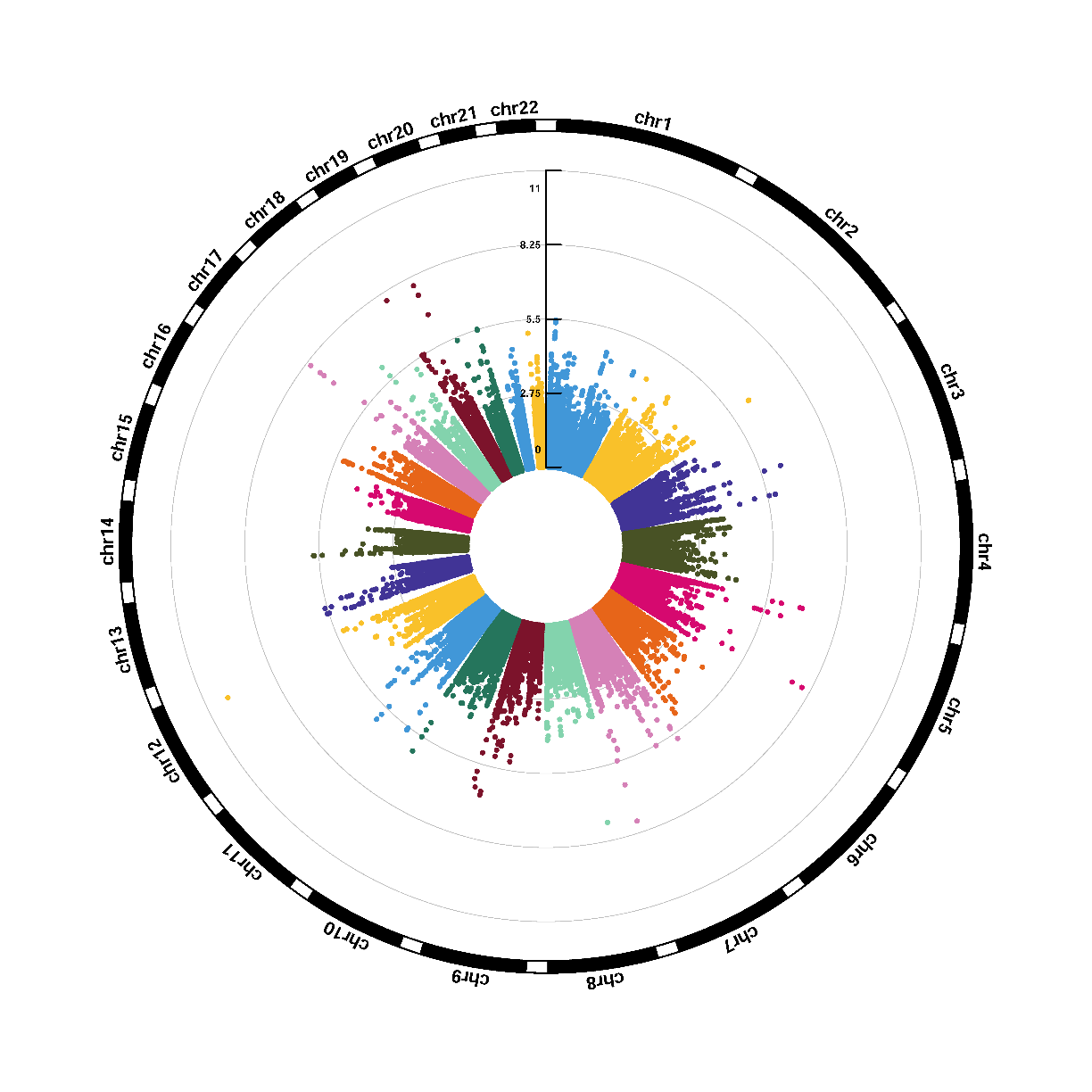


**Additional file 2: Fig. S1**. Circular Manhattan plot for epigenome-wide association study on fasting plasma glucose. The numbers of chromosome and the -log_10_ of *P*-values for statistical significance are shown. Dots represent the observed CpGs.
