# Supplementary material for: Identification of key DNA methylation changes on fasting plasma glucose: a genome-wide DNA methylation analysis in Chinese monozygotic twins
Source: Diabetol Metab Syndr. 2023 Jul 17;15:159. doi: 10.1186/s13098-023-01136-4 (PMC10351111; doi:10.1186/s13098-023-01136-4)
Supplement: Supplementary file 3 — Additional file 3: Table S2. The top GREAT ontology enrichments potentially related to fasting plasma glucose using binomial test. [file 13098_2023_1136_MOESM3_ESM.docx]

**Additional file 3: Table S2**. The top GREAT ontology enrichments potentially related to fasting plasma glucose using binomial test.

| **Ontology database** | **Term name** | **Binom FDR Q-Value** | **Binom region fold enrichment** |
| --- | --- | --- | --- |
| ***GO function*** | | | |
| GO-MF | Sequence-specific DNA binding | 2.27E-91 | 1.47 |
| GO-MF | Nucleic acid binding | 3.07E-85 | 1.22 |
| GO-MF | Phospholipid-hydroperoxide glutathione peroxidase activity | 1.25E-40 | 57.00 |
| GO-MF | Mitogen-activated protein kinase p38 binding | 3.76E-40 | 8.88 |
| GO-MF | Platelet-derived growth factor binding | 1.24E-35 | 4.01 |
| GO-MF | Enhancer binding | 1.05E-28 | 2.19 |
| GO-MF | Telomerase activity | 1.62E-23 | 13.10 |
| GO-MF | Metal ion binding | 9.34E-21 | 1.09 |
| GO-MF | FK506 binding | 1.62E-19 | 3.75 |
| GO-BP | Positive regulation of insulin receptor signaling pathway | 1.83E-61 | 7.80 |
| GO-BP | Regulation of biosynthetic process | 4.68E-54 | 1.15 |
| GO-BP | Cell fate commitment | 5.26E-47 | 1.54 |
| GO-BP | Regulation of gene expression | 2.14E-46 | 1.14 |
| GO-BP | White fat cell differentiation | 1.18E-45 | 4.22 |
| GO-BP | Regulation of biological process | 1.75E-43 | 1.06 |
| GO-BP | Cortisol biosynthetic process | 1.16E-42 | 21.40 |
| GO-BP | Aldosterone biosynthetic process | 1.16E-42 | 21.40 |
| Human Phenotype | Intrauterine growth retardation | 7.49E-26 | 1.56 |
| Disease Ontology | Acanthosis nigricans | 8.42E-35 | 33.90 |
| Disease Ontology | Ulcerative colitis | 7.28E-16 | 1.50 |
| Disease Ontology | Williams syndrome | 7.26E-14 | 4.66 |
| Disease Ontology | Tangier disease | 2.62E-12 | 5.08 |
| ***Pathways*** | | | |
| PANTHER | Notch signaling pathway | 1.98E-33 | 2.66 |
| PANTHER | Huntington disease | 1.01E-22 | 1.69 |
| PANTHER | Circadian clock system | 1.33E-11 | 3.59 |
| PANTHER | Adrenaline and noradrenaline biosynthesis | 2.94E-10 | 2.19 |
| PANTHER | Nicotine pharmacodynamics pathway | 9.41E-07 | 1.78 |
| PANTHER | Nicotinic acetylcholine receptor signaling pathway | 2.81E-05 | 1.38 |
| PANTHER | Wnt signaling pathway | 6.82E-05 | 1.15 |
| PANTHER | Beta3 adrenergic receptor signaling pathway | 1.91E-03 | 1.56 |
| PANTHER | Inflammation mediated by chemokine and cytokine Signaling pathway | 2.51E-03 | 1.18 |
| PANTHER | Gamma-aminobutyric acid synthesis | 3.95E-03 | 2.34 |
| BioCyc | Chondroitin sulfate degradation | 4.38E-10 | 4.52 |
| BioCyc | Methylglyoxal degradation VI | 4.19E-08 | 3.19 |
| BioCyc | Palmitate biosynthesis I | 5.52E-03 | 2.18 |
| BioCyc | Gluconeogenesis | 5.35E-03 | 1.63 |
| MSigDB | Notch signaling pathway | 1.85E-43 | 3.05 |
| MSigDB | Glucagon signaling in metabolic regulation | 2.01E-17 | 2.29 |
| MSigDB | Biosynthesis of neurotransmitters | 1.73E-15 | 6.46 |
| MSigDB | Incretin synthesis, secretion, and inactivation | 9.37E-15 | 2.33 |
| MSigDB | Glucagon-type ligand receptors | 7.48E-14 | 2.37 |
| MSigDB | Synthesis, secretion, and inactivation of glucose-dependent insulinotropic polypeptide (GIP) | 3.92E-11 | 2.39 |
| MSigDB | Endocytosis | 4.64E-11 | 1.40 |
| MSigDB | Regulation of insulin-like growth factor (IGF) activity by insulin-like Growth factor binding proteins (IGFBPs) | 9.21E-09 | 2.17 |
| MSigDB | Steroid biosynthesis | 4.99E-05 | 2.17 |

**Note:** BP, biological process; FDR, false discovery rate; MF, molecular function.
