# Supplementary material for: Identification of key DNA methylation changes on fasting plasma glucose: a genome-wide DNA methylation analysis in Chinese monozygotic twins
Source: Diabetol Metab Syndr. 2023 Jul 17;15:159. doi: 10.1186/s13098-023-01136-4 (PMC10351111; doi:10.1186/s13098-023-01136-4)
Supplement: Supplementary file 4 — Additional file 4: Table S3. The results of DNA methylation of top 30 CpGs with fasting plasma glucose in epigenome-wide association analysis in sensitivity analysis that further adjusting for smoking status and drinking status in GEE model. [file 13098_2023_1136_MOESM4_ESM.docx]

**Additional file 4: Table S3**. The results of DNA methylation of top 30 CpGs with fasting plasma glucose in epigenome-wide association analysis in sensitivity analysis that further adjusting for smoking status and drinking status in GEE model.

| **CpG No.** | **Chromosome** | **Position (bp)** | ***β*** | ***P*-value** | **FDR** | **HGNC symbol** |
| --- | --- | --- | --- | --- | --- | --- |
| 1 | chr5 | 150,027,611 | 0.264 | 1.11E-10 | 2.81E-05 | *SYNPO* |
| 2 | chr12 | 105,478,501 | 2.474 | 3.09E-10 | 3.91E-05 | *ALDH1L2* |
| 3 | chr5 | 150,027,616 | 0.254 | 6.93E-10 | 5.84E-05 | *SYNPO* |
| 4 | chr19 | 658,314 | 0.326 | 1.07E-08 | 6.73E-04 | *RNF126* |
| 5 | chr8 | 26,148,178 | -1.786 | 1.77E-08 | 8.96E-04 | *PPP2R2A* |
| 6 | chr19 | 59,073,819 | 0.304 | 2.35E-08 | 9.88E-04 | *MZF1* |
| 7 | chr17 | 27,052,829 | 2.723 | 4.29E-08 | 1.40E-03 | *TLCD1* |
| 8 | chr9 | 133,911,755 | 0.126 | 4.78E-08 | 1.40E-03 | *LAMC3* |
| 9 | chr19 | 59,073,806 | 0.257 | 4.98E-08 | 1.40E-03 | *MZF1* |
| 10 | chr17 | 27,052,816 | 2.718 | 6.01E-08 | 1.51E-03 | *TLCD1* |
| 11 | chr17 | 27,052,798 | 2.781 | 6.60E-08 | 1.51E-03 | *TLCD1* |
| 12 | chr5 | 29,364,034 | 0.241 | 1.18E-07 | 2.47E-03 | *LINC02064* |
| 13 | chr7 | 157,670,224 | 0.224 | 1.68E-07 | 2.89E-03 | *PTPRN2* |
| 14 | chr9 | 119,332,867 | 0.318 | 1.71E-07 | 2.89E-03 | *ASTN2* |
| 15 | chr17 | 27,052,771 | 2.782 | 1.71E-07 | 2.89E-03 | *TLCD1* |
| 16 | chr9 | 140,033,560 | -0.273 | 3.32E-07 | 5.25E-03 | *GRIN1* |
| 17 | chr5 | 29,364,022 | 0.224 | 4.48E-07 | 6.65E-03 | *LINC02064* |
| 18 | chr11 | 72,352,936 | 0.231 | 5.10E-07 | 7.14E-03 | *PDE2A* |
| 19 | chr19 | 59,073,831 | 0.331 | 5.39E-07 | 7.14E-03 | *MZF1* |
| 20 | chr9 | 96,009,797 | -2.118 | 5.65E-07 | 7.14E-03 | *WNK2* |
| 21 | chr10 | 126,490,028 | 0.174 | 5.96E-07 | 7.16E-03 | *FAM175B* |
| 22 | chr9 | 140,033,557 | -0.262 | 6.42E-07 | 7.37E-03 | *GRIN1* |
| 23 | chr9 | 119,332,835 | 0.294 | 7.31E-07 | 8.03E-03 | *ASTN2* |
| 24 | chr3 | 189,831,588 | 0.411 | 8.67E-07 | 9.00E-03 | *LEPREL1* |
| 25 | chr5 | 1,233,066 | 0.174 | 9.36E-07 | 9.00E-03 | *SLC6A18* |
| 26 | chr5 | 1,233,041 | 0.204 | 9.93E-07 | 9.00E-03 | *SLC6A18* |
| 27 | chr5 | 1,233,045 | 0.199 | 9.96E-07 | 9.00E-03 | *SLC6A18* |
| 28 | chr3 | 129,059,046 | -2.500 | 1.00E-06 | 9.00E-03 | *MARK2P19* |
| 29 | chr5 | 1,233,035 | 0.208 | 1.03E-06 | 9.00E-03 | *SLC6A18* |
| 30 | chr19 | 658,287 | 0.294 | 1.10E-06 | 9.26E-03 | *RNF126* |

**Note**: FDR, false discovery rate; *β*, regression coefficient.
