# Supplementary material for: Identification of key DNA methylation changes on fasting plasma glucose: a genome-wide DNA methylation analysis in Chinese monozygotic twins
Source: Diabetol Metab Syndr. 2023 Jul 17;15:159. doi: 10.1186/s13098-023-01136-4 (PMC10351111; doi:10.1186/s13098-023-01136-4)
Supplement: Supplementary file 5 — Additional file 5: Table S4. The results of annotation to significant differentially methylated regions for fasting plasma glucose in sensitivity analysis. [file 13098_2023_1136_MOESM5_ESM.docx]

**Additional file 5: Table S4**. The results of annotation to significant differentially methylated regions for fasting plasma glucose in sensitivity analysis.

| **DMR No.** | **Chromosome** | **Start** | **End** | **Length** | ***slk* corrected *P*-value** | **Gene symbol** |
| --- | --- | --- | --- | --- | --- | --- |
| 1 | chr17 | 27,052,679 | 27,052,830 | 6 | 1.97E-05 | *TLCD1* |
| 2 | chr13 | 52,769,630 | 52,769,786 | 11 | 2.12E-04 | *MRPS31P5* |
| 3 | chr5 | 29,364,017 | 29,364,153 | 11 | 2.48E-04 | NA |
| 4 | chr11 | 1,985,750 | 1,985,907 | 10 | 2.74E-04 | *MRPL23* |
| 5 | chr18 | 14,458,531 | 14,458,993 | 25 | 3.46E-04 | *CXADRP3* |
| 6 | chr11 | 1,989,899 | 1,991,115 | 33 | 4.91E-04 | *AK126380* |
| 7 | chr9 | 44,118,236 | 44,118,478 | 21 | 1.38E-03 | NA |
| 8 | chr1 | 155,790,779 | 155,790,915 | 11 | 1.48E-03 | *GON4L* |
| 9 | chr7 | 157,670,041 | 157,670,346 | 15 | 1.90E-03 | *PTPRN2* |
| 10 | chr17 | 35,017,806 | 35,017,947 | 11 | 2.18E-03 | NA |
| 11 | chr5 | 150,027,514 | 150,027,745 | 10 | 2.71E-03 | *SYNPO* |
| 12 | chr1 | 10,718,376 | 10,718,662 | 12 | 2.71E-03 | *CASZ1* |
| 13 | chr17 | 36,413,806 | 36,413,942 | 12 | 4.32E-03 | *LOC440434* |
| 14 | chr1 | 3,111,455 | 3,111,772 | 20 | 5.24E-03 | *PRDM16* |
| 15 | chr1 | 180,922,670 | 180,923,856 | 41 | 7.34E-03 | *AK056657* |
| 16 | chr16 | 33,509,364 | 33,509,939 | 41 | 8.43E-03 | NA |
| 17 | chr18 | 74,154,009 | 74,154,636 | 31 | 8.87E-03 | *ZNF516* |
| 18 | chr11 | 396,866 | 397,176 | 17 | 9.37E-03 | *PKP3* |
| 19 | chr1 | 53,904,769 | 53,904,934 | 15 | 1.12E-02 | *SLC25A3P1* |
| 20 | chr22 | 38,723,759 | 38,724,067 | 13 | 1.21E-02 | *CSNK1E* |
| 21 | chr17 | 20,747,104 | 20,747,489 | 38 | 1.66E-02 | *BC067347* |
| 22 | chr19 | 37,329,666 | 37,329,802 | 11 | 1.71E-02 | *ZNF790* |
| 23 | chr16 | 86,587,026 | 86,587,188 | 8 | 1.88E-02 | *MTHFSD* |
| 24 | chr17 | 20,658,746 | 20,658,885 | 11 | 1.98E-02 | NA |
| 25 | chr7 | 56,439,560 | 56,439,713 | 11 | 2.14E-02 | NA |
| 26 | chr10 | 126,489,968 | 126,490,164 | 9 | 2.28E-02 | *FAM175B* |
| 27 | chr14 | 37,667,220 | 37,667,455 | 16 | 2.50E-02 | *MIPOL1* |
| 28 | chr12 | 34,499,816 | 34,501,260 | 55 | 2.79E-02 | NA |
| 29 | chr8 | 1,497,065 | 1,497,187 | 13 | 2.85E-02 | *DLGAP2* |
| 30 | chr19 | 36,303,282 | 36,303,702 | 19 | 2.89E-02 | *PRODH2* |
| 31 | chr22 | 32,598,662 | 32,599,407 | 26 | 2.95E-02 | *RFPL2* |
| 32 | chr4 | 2,765,396 | 2,765,769 | 21 | 3.07E-02 | *TNIP2* |
| 33 | chr4 | 11,552,956 | 11,553,084 | 11 | 3.85E-02 | NA |
| 34 | chr1 | 30,758,193 | 30,758,450 | 15 | 4.05E-02 | NA |
| 35 | chr18 | 55,103,227 | 55,104,186 | 60 | 4.06E-02 | *ONECUT2* |
| 36 | chr19 | 14,089,842 | 14,090,122 | 11 | 4.32E-02 | *RFX1* |
| 37 | chr18 | 73,176 | 73,654 | 32 | 4.34E-02 | *TUBB8B* |
| 38 | chr13 | 20,138,762 | 20,139,122 | 23 | 4.66E-02 | *TPTE2-AS1* |
| 39 | chr4 | 30,724,012 | 30,724,138 | 16 | 4.80E-02 | *PCDH7* |
| 40 | chr7 | 15,725,475 | 15,725,592 | 13 | 4.87E-02 | *MEOX2* |

**Note:** DMR, differentially methylated region; NA, not available.
