# Supplementary material for: Identification of key DNA methylation changes on fasting plasma glucose: a genome-wide DNA methylation analysis in Chinese monozygotic twins
Source: Diabetol Metab Syndr. 2023 Jul 17;15:159. doi: 10.1186/s13098-023-01136-4 (PMC10351111; doi:10.1186/s13098-023-01136-4)
Supplement: Supplementary file 6 — Additional file 6: Table S5. The results of causal inference analysis of top CpGs with fasting plasma glucose in sensitivity analysis. [file 13098_2023_1136_MOESM6_ESM.docx]

**Additional file 6: Table S5**. The results of causal inference analysis of top CpGs with fasting plasma glucose in sensitivity analysis.

| **Chromosome** | **Position (bp)** | **HGNC symbol** | **Methylation to FPG** | | | | |  | **FPG to methylation** | | | | |
| --- | --- | --- | --- | --- | --- | --- | --- | --- | --- | --- | --- | --- | --- |
|  |  |  | *β*_self_ change_ | *P*_self_ change_ | *β*_co-twin_ change_ | *P*_co-twin_ change_ | Absolute value of ratio |  | *β*_self_ change_ | *P*_self_ change_ | *β*_co-twin_ change_ | *P*_co-twin_ change_ | Absolute value of ratio |
| chr5 | 150,027,611 | *SYNPO* | 0.082 | 1.23E-03 | 0.105 | 2.20E-03 | 1.283 |  | -0.024 | 6.25E-01 | -0.049 | 2.60E-01 | 2.090 |
| chr12 | 105,478,501 | *ALDH1L2* | 0.016 | 4.63E-10 | 0.017 | 2.89E-10 | 1.065 |  | -0.500 | 3.13E-01 | -0.645 | 2.10E-01 | 1.290 |
| chr5 | 150,027,616 | *SYNPO* | 0.075 | 3.25E-03 | 0.095 | 4.42E-03 | 1.257 |  | -0.023 | 6.43E-01 | -0.044 | 3.23E-01 | 1.936 |
| chr19 | 658,314 | *RNF126* | 0.046 | 1.41E-03 | 0.068 | 1.86E-06 | 1.472 |  | -0.023 | 6.84E-01 | -0.170 | 1.56E-01 | 7.489 |
| chr8 | 26,148,178 | *PPP2R2A* | -0.017 | 7.86E-05 | -0.011 | 2.22E-03 | 0.618 |  | 0.630 | 1.23E-01 | 0.133 | 7.52E-01 | 0.212 |
| chr19^§^ | 59,073,819 | *MZF1* | **0.029** | **3.96E-01** | **0.151** | **9.45E-03** | **5.230** |  | 0.018 | 7.19E-01 | -0.114 | 7.82E-02 | 6.428 |
| chr17^#^ | 27,052,829 | *TLCD1* | 0.017 | 2.03E-06 | 0.024 | 3.94E-05 | 1.421 |  | **-0.329** | **5.35E-01** | **-1.059** | **1.90E-02** | **3.218** |
| chr9 | 133,911,755 | *LAMC3* | -0.030 | 5.49E-01 | 0.088 | 1.59E-01 | 2.905 |  | 0.018 | 6.28E-01 | -0.029 | 3.65E-01 | 1.556 |
| chr19^§^ | 59,073,806 | *MZF1* | **0.022** | **5.99E-01** | **0.112** | **4.71E-02** | **5.141** |  | 0.010 | 8.42E-01 | -0.080 | 1.76E-01 | 7.954 |
| chr17^#^ | 27,052,816 | *TLCD1* | 0.016 | 7.29E-05 | 0.023 | 5.33E-04 | 1.449 |  | **-0.366** | **4.68E-01** | **-1.077** | **1.80E-02** | **2.944** |
| chr17^#^ | 27,052,798 | *TLCD1* | 0.014 | 6.92E-04 | 0.019 | 2.95E-03 | 1.345 |  | **-0.448** | **3.91E-01** | **-0.963** | **4.57E-02** | **2.149** |
| chr5 | 29,364,034 | *LINC02064* | 0.044 | 3.14E-02 | 0.062 | 2.76E-03 | 1.419 |  | -0.030 | 6.24E-01 | -0.145 | 2.55E-01 | 4.921 |
| chr7^§^ | 157,670,224 | *PTPRN2* | **0.077** | **3.46E-06** | **0.149** | **2.03E-09** | **1.931** |  | 0.001 | 9.81E-01 | -0.072 | 1.12E-01 | 71.878 |
| chr9^#^ | 119,332,867 | *ASTN2* | 0.154 | 2.04E-06 | 0.204 | 9.23E-08 | 1.331 |  | **-0.044** | **4.75E-01** | **-0.159** | **3.81E-03** | **3.636** |
| chr17 | 27,052,771 | *TLCD1* | 0.013 | 5.05E-03 | 0.015 | 1.54E-02 | 1.159 |  | -0.568 | 3.03E-01 | -0.797 | 1.41E-01 | 1.404 |
| chr9^§^ | 140,033,560 | *GRIN1* | **0.029** | **2.49E-01** | **-0.047** | **4.72E-02** | **1.618** |  | -0.055 | 2.76E-01 | 0.064 | 1.29E-01 | 1.165 |
| chr5 | 29,364,022 | *LINC02064* | 0.043 | 8.20E-02 | 0.056 | 6.55E-03 | 1.313 |  | -0.043 | 4.56E-01 | -0.124 | 3.12E-01 | 2.860 |
| chr11^§#^ | 72,352,936 | *PDE2A* | **0.143** | **1.95E-06** | **0.253** | **5.51E-10** | **1.770** |  | **-0.006** | **8.45E-01** | **-0.088** | **4.89E-03** | **14.239** |
| chr19^§^ | 59,073,831 | *MZF1* | **0.025** | **4.45E-01** | **0.150** | **4.51E-03** | **6.074** |  | 0.024 | 6.57E-01 | -0.130 | 8.19E-02 | 5.416 |
| chr9^§#^ | 96,009,797 | *WNK2* | **-0.016** | **1.85E-06** | **-0.029** | **2.45E-07** | **1.832** |  | **0.029** | **9.34E-01** | **1.176** | **8.74E-03** | **40.608** |
| chr10 | 126,490,028 | *FAM175B* | -0.036 | 6.57E-01 | 0.209 | 9.03E-02 | 5.886 |  | 0.010 | 7.41E-01 | -0.028 | 4.45E-01 | 2.713 |
| chr9^§^ | 140,033,557 | *GRIN1* | **0.030** | **2.29E-01** | **-0.047** | **4.69E-02** | **1.548** |  | -0.053 | 2.72E-01 | 0.060 | 1.39E-01 | 1.148 |
| chr9^#^ | 119,332,835 | *ASTN2* | 0.091 | 1.65E-03 | 0.129 | 1.24E-05 | 1.425 |  | **-0.047** | **4.56E-01** | **-0.121** | **3.26E-02** | **2.598** |
| chr3^§^ | 189,831,588 | *LEPREL1* | **0.057** | **6.29E-03** | **0.087** | **8.76E-03** | **1.529** |  | -0.044 | 5.28E-01 | -0.133 | 7.72E-02 | 3.015 |
| chr5^§#^ | 1,233,066 | *SLC6A18* | **0.091** | **4.87E-06** | **0.177** | **2.09E-10** | **1.950** |  | **0.003** | **9.41E-01** | **-0.070** | **3.82E-02** | **25.124** |
| chr5^§#^ | 1,233,041 | *SLC6A18* | **0.134** | **8.86E-08** | **0.250** | **9.29E-09** | **1.861** |  | **0.008** | **8.17E-01** | **-0.093** | **1.18E-02** | **11.256** |
| chr5^§#^ | 1,233,045 | *SLC6A18* | **0.134** | **1.61E-07** | **0.249** | **1.14E-08** | **1.867** |  | **0.009** | **8.00E-01** | **-0.092** | **1.09E-02** | **10.206** |
| chr3^§^ | 129,059,046 | *MARK2P19* | **-0.001** | **9.16E-01** | **-0.021** | **6.60E-03** | **41.612** |  | -0.326 | 4.63E-01 | 0.935 | 5.72E-02 | 2.869 |
| chr5^§#^ | 1,233,035 | *SLC6A18* | **0.140** | **7.45E-09** | **0.253** | **1.90E-09** | **1.808** |  | **0.005** | **8.90E-01** | **-0.091** | **1.38E-02** | **18.774** |
| chr19 | 658,287 | *RNF126* | 0.020 | 3.06E-01 | 0.049 | 6.25E-02 | 2.437 |  | 0.012 | 8.42E-01 | -0.159 | 2.09E-01 | 13.338 |

**Note**: FPG, fasting plasma glucose.

^§^ The change of DNA methylation causes the FPG change.

^#^ The FPG change causes the change of DNA methylation.
