# Supplementary material for: Identification of key DNA methylation changes on fasting plasma glucose: a genome-wide DNA methylation analysis in Chinese monozygotic twins
Source: Diabetol Metab Syndr. 2023 Jul 17;15:159. doi: 10.1186/s13098-023-01136-4 (PMC10351111; doi:10.1186/s13098-023-01136-4)
Supplement: Supplementary file 7 — Additional file 7: Table S6. Basic characteristics of participants from the community in the quantitative methylation analysis of SLC6A18 gene [file 13098_2023_1136_MOESM7_ESM.docx]

**Additional file 7: Table S6**. Basic characteristics of participants from the community in the quantitative methylation analysis of SLC6A18 gene.

| **Characteristics** | **Non-diabetes** | **Diabetes** | ***P*-value** |
| --- | --- | --- | --- |
|  | (n = 170) | (n = 72) |  |
| Sex (%) |  |  |  |
| Male | 82 (48.24) | 34 (47.22) | 0.997 |
| Female | 88 (51.76) | 38 (52.78) |  |
| Age, years | 52 (37.28, 72.73) | 56.26 (11.70) | 0.058 |
| BMI, kg/m^2^ | 24.89 (18.90, 33.34) | 25.14 (2.17) | 0.463 |
| Systolic, mmHg | 128.50 (104.28, 180.45) | 131.33 (105.50, 165.26) | 0.390 |
| Diastolic, mmHg | 80.00 (64.00, 104.35) | 80.49 (9.48) | 0.391 |
| FPG, mmol/L | 5.12 (0.76) | 8.77 (7.07, 19.95) | < 0.001 |
| CHOL, mmol/L | 4.93 (1.06) | 5.51 (1.23) | < 0.001 |
| TG, mmol/L | 1.15 (0.37, 3.94) | 2.04 (0.63, 9.23) | < 0.001 |
| HDLC, mmol/L | 1.43 (0.78, 2.58) | 1.38 (0.36) | 0.050 |
| LDLC, mmol/L | 2.34 (1.28, 4.28) | 2.14 (0.66) | 0.001 |

**Note:** Continuous variables were presented as mean (standard deviation (SD)) or median (P_2.5_, P_97.5_); Categorical variables were presented as numbers with percentages. BMI, body mass index; CHOL, total cholesterol; FPG, fasting plasma glucose; HDLC, high-density lipoprotein cholesterol; LDLC, low-density lipoprotein cholesterol; SUA, serum uric acid; TG, triglyceride
