# Supplementary material for: Identification of key DNA methylation changes on fasting plasma glucose: a genome-wide DNA methylation analysis in Chinese monozygotic twins
Source: Diabetol Metab Syndr. 2023 Jul 17;15:159. doi: 10.1186/s13098-023-01136-4 (PMC10351111; doi:10.1186/s13098-023-01136-4)
Supplement: Supplementary file 8 — Additional file 8: Table S7. Validation analysis results for the CpGs mapped to SLC6A18 gene. [file 13098_2023_1136_MOESM8_ESM.docx]

**Additional file 8: Table S7**. Validation analysis results for the CpGs mapped to *SLC6A18* gene.

| **CpG No.** | **Chromosome** | **Position (bp)** | **Discovery** | |  | **Validation** | | | | |
| --- | --- | --- | --- | --- | --- | --- | --- | --- | --- | --- |
|  |  |  | *β* | *P*-value |  | *P*-value of comparison between groups |  | *β* | *P*-value | OR (95% CI) |
| 1 | chr5 | 1,233,066 | 0.181 | 1.61E-07 |  | < 0.001 |  | 0.216 | 0.033 | 1.241 (1.022-1.524) |
| 2 | chr5 | 1,233,089 | 0.121 | 4.08E-04 |  | < 0.001 |  | 0.371 | 0.037 | 1.448 (1.025-2.068) |
| 3 | chr5 | 1,233,096 | 0.097 | 9.08E-03 |  | < 0.001 |  | 0.355 | 0.046 | 1.426 (1.009-2.035) |

**Note**: *β*, regression coefficient.
