# Supplementary material for: Identification of key DNA methylation changes on fasting plasma glucose: a genome-wide DNA methylation analysis in Chinese monozygotic twins
Source: Diabetol Metab Syndr. 2023 Jul 17;15:159. doi: 10.1186/s13098-023-01136-4 (PMC10351111; doi:10.1186/s13098-023-01136-4)
Supplement: Supplementary file 9 — Additional file 9: Figure S2. Relationships between consensus module eigengenes and external trait. Each row in the table corresponds to a consensus module, and each column to a trait. Numbers in the table report the correlations of the corresponding module eigengenes and trait with the P-values printed below the correlations in parentheses. The table is color coded by correlation according to the shade of color legend. FPG, fasting plasma glucose. [file 13098_2023_1136_MOESM9_ESM.docx]

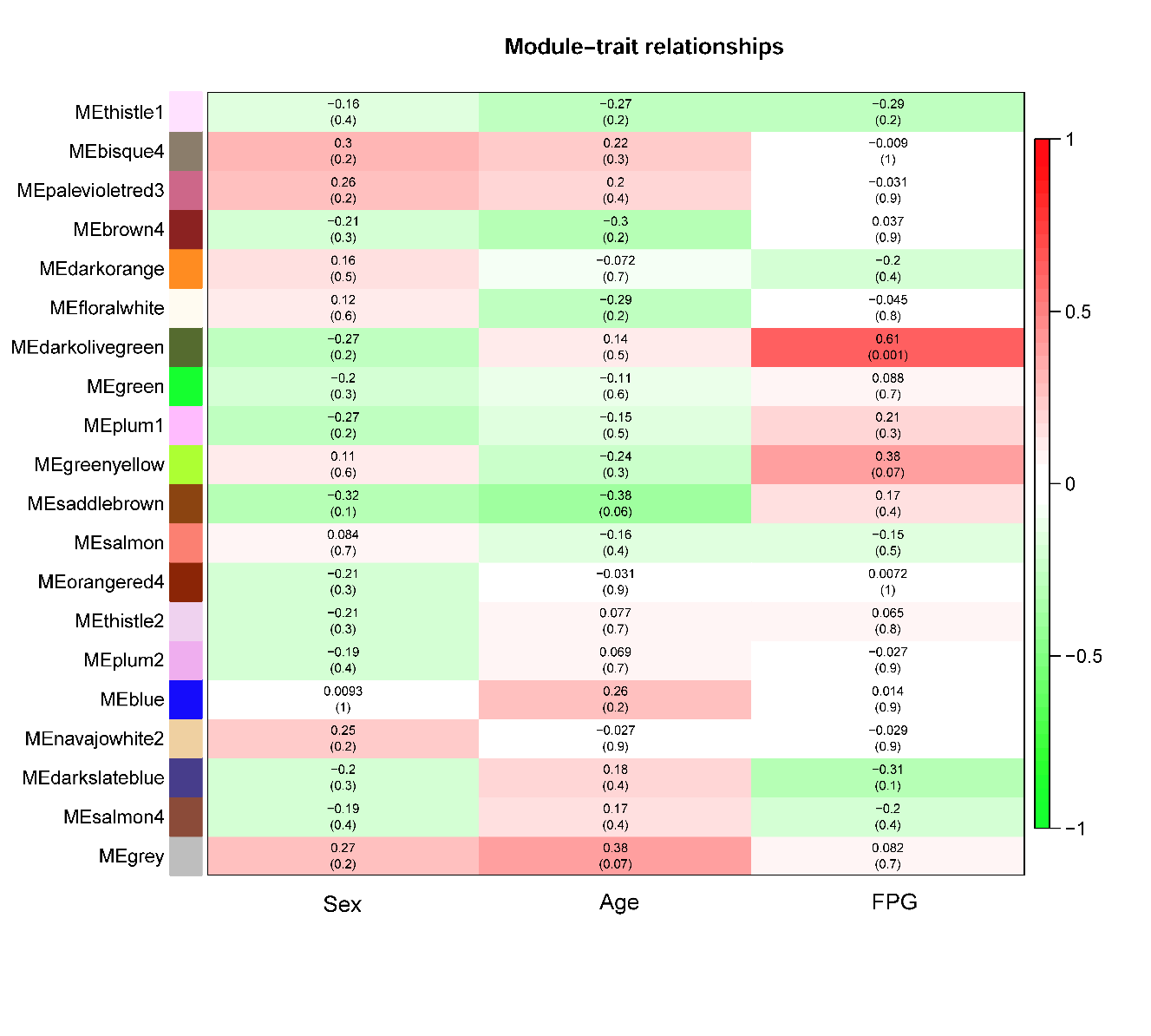


**Additional file 9: Fig. S2**. Relationships between consensus module eigengenes and external trait. Each row in the table corresponds to a consensus module, and each column to a trait. Numbers in the table report the correlations of the corresponding module eigengenes and trait with the *P*-values printed below the correlations in parentheses. The table is color coded by correlation according to the shade of color legend. FPG, fasting plasma glucose.
