# Supplementary material for: Identification of key DNA methylation changes on fasting plasma glucose: a genome-wide DNA methylation analysis in Chinese monozygotic twins
Source: Diabetol Metab Syndr. 2023 Jul 17;15:159. doi: 10.1186/s13098-023-01136-4 (PMC10351111; doi:10.1186/s13098-023-01136-4)
Supplement: Supplementary file 10 — Additional file 10: Table S8. The common genes between DNA methylation analysis and gene expression analysis. [file 13098_2023_1136_MOESM10_ESM.docx]

**Additional file 10: Table S8**. The common genes between DNA methylation analysis and gene expression analysis.

| **Ensembl gene ID** | **HGNC symbol** | **Genes where the top CpGs (*P <* 1×10^-6^) located** | **Genes where the DMRs located** | **Genes identified by causal inference** |
| --- | --- | --- | --- | --- |
| ENSG00000136010 | *ALDH1L2* | yes | yes |  |
| ENSG00000160606 | *TLCD1* | yes | yes | yes |
| ENSG00000070423 | *RNF126* | yes |  |  |
| ENSG00000171992 | *SYNPO* | yes | yes |  |
| ENSG00000099326 | *MZF1* | yes |  | yes |
| ENSG00000155093 | *PTPRN2※* | yes | yes | yes |
| ENSG00000221914 | *PPP2R2A* | yes |  |  |
| ENSG00000164363 | *SLC6A18* | yes |  | yes |
| ENSG00000148219 | *ASTN2* | yes |  | yes |
| ENSG00000050555 | *LAMC3* | yes |  |  |
| ENSG00000132321 | *IQCA1* | yes |  | yes |
| ENSG00000165660 | *FAM175B* | yes | yes |  |
| ENSG00000176884 | *GRIN1* | yes |  | yes |
| ENSG00000186642 | *PDE2A* | yes |  | yes |
| ENSG00000090530 | *P3H2* |  |  |  |
| ENSG00000146540 | *C7orf50※* |  |  |  |
| ENSG00000100918 | *REC8* |  |  |  |
| ENSG00000214026 | *MRPL23* |  | yes |  |
| ENSG00000120693 | *SMAD9* |  |  |  |
| ENSG00000170419 | *VSTM2A* |  |  |  |
| ENSG00000130589 | *HELZ2* |  |  |  |
| ENSG00000105428 | *ZNRF4* |  |  |  |
| ENSG00000130940 | *CASZ1※* |  | yes |  |
| ENSG00000125845 | *BMP2* |  |  |  |
| ENSG00000251692 | *PTX4* |  |  |  |
| ENSG00000112787 | *FBRSL1* |  |  |  |
| ENSG00000164828 | *SUN1* |  |  |  |
| ENSG00000122390 | *NAA60* |  |  |  |
| ENSG00000174469 | *CNTNAP2* |  |  |  |
| ENSG00000161992 | *PRR35* |  |  |  |
| ENSG00000182902 | *SLC25A18* |  |  |  |
| ENSG00000141564 | *RPTOR* |  |  |  |
| ENSG00000196967 | *ZNF585A* |  |  |  |
| ENSG00000170276 | *HSPB2* |  |  |  |
| ENSG00000065000 | *AP3D1* |  |  |  |
| ENSG00000125337 | *KIF25* |  |  |  |
| ENSG00000130702 | *LAMA5* |  |  |  |
| ENSG00000006194 | *ZNF263* |  |  |  |
| ENSG00000171612 | *SLC25A33* |  |  |  |
| ENSG00000196498 | *NCOR2* |  |  |  |
| ENSG00000109846 | *CRYAB* |  |  |  |
| ENSG00000122691 | *TWIST1* |  |  |  |
| ENSG00000166984 | *TCP10L2* |  |  |  |
| ENSG00000130383 | *FUT5* |  |  |  |
| ENSG00000165238 | *WNK2* |  |  |  |
| ENSG00000116580 | *GON4L* |  | yes |  |
| ENSG00000152495 | *CAMK4* |  |  |  |
| ENSG00000188833 | *ENTPD8* |  |  |  |
| ENSG00000120256 | *LRP11* |  |  |  |
| ENSG00000141556 | *TBCD* |  |  |  |
| ENSG00000129521 | *EGLN3* |  |  |  |
| ENSG00000160299 | *PCNT* |  |  |  |
| ENSG00000154040 | *CABYR* |  |  |  |
| ENSG00000197724 | *PHF2* |  |  |  |
| ENSG00000062038 | *CDH3* |  |  |  |
| ENSG00000187475 | *HIST1H1T* |  |  |  |
| ENSG00000140398 | *NEIL1* |  |  |  |
| ENSG00000244274 | *DBNDD2* |  |  |  |
| ENSG00000124251 | *TP53TG5* |  |  |  |
| ENSG00000120332 | *TNN* |  |  |  |
| ENSG00000153303 | *FRMD1* |  |  |  |
| ENSG00000115756 | *HPCAL1* |  |  |  |
| ENSG00000014164 | *ZC3H3* |  |  |  |
| ENSG00000168310 | *IRF2* |  |  |  |
| ENSG00000072071 | *ADGRL1* |  |  |  |
| ENSG00000011485 | *PPP5C* |  |  |  |
| ENSG00000166106 | *ADAMTS15* |  |  |  |
| ENSG00000157933 | *SKI※* |  |  |  |
| ENSG00000172382 | *PRSS27* |  |  |  |
| ENSG00000142279 | *WTIP* |  |  |  |
| ENSG00000162572 | *SCNN1D* |  |  |  |
| ENSG00000168779 | *SHOX2* |  |  |  |
| ENSG00000163637 | *PRICKLE2* |  |  |  |
| ENSG00000162105 | *SHANK2* |  |  |  |
| ENSG00000213923 | *CSNK1E* |  | yes |  |
| ENSG00000127666 | *TICAM1* |  |  |  |
| ENSG00000164930 | *FZD6* |  |  |  |
| ENSG00000184967 | *NOC4L* |  |  |  |
| **Note:** DMRs, differentially methylated regions.  ※Genes already suggested by previous studies. | | | | |
