# Supplementary material for: Identification of key DNA methylation changes on fasting plasma glucose: a genome-wide DNA methylation analysis in Chinese monozygotic twins
Source: Diabetol Metab Syndr. 2023 Jul 17;15:159. doi: 10.1186/s13098-023-01136-4 (PMC10351111; doi:10.1186/s13098-023-01136-4)
Supplement: Supplementary file 11 — Additional file 11: Table S9. The common biological enrichment terms between DNA methylation analysis and gene expression analysis. [file 13098_2023_1136_MOESM11_ESM.docx]

**Additional file 11: Table S9**. The common biological enrichment terms between DNA methylation analysis and gene expression analysis.

| **Ontology database** | **ID** | **Term name** |
| --- | --- | --- |
| GO-MF | GO:0005201 | Extracellular matrix structural constituent |
| GO-MF | GO:0035240 | Dopamine binding |
| GO-MF | GO:0004952 | Dopamine neurotransmitter receptor activity |
| GO-BP | GO:0009889 | Regulation of biosynthetic process |
| GO-BP | GO:0048665 | Neuron fate specification |
| GO-BP | GO:0045165 | Cell fate commitment |
| GO-BP | GO:0021516 | Dorsal spinal cord development |
| GO-MF | GO:0005549 | Odorant binding |
| GO-BP | GO:0021527 | Spinal cord association neuron differentiation |
| GO-BP | GO:0009953 | Dorsal/ventral pattern formation |
| GO-BP | GO:0006700 | C21-steroid hormone biosynthetic process |

**Note**: BP, biological process; FDR, false discovery rate; MF, molecular function.
